# Different circadian patterns of arterial stiffness are responsible for increased cardiovascular mortality in hemodialyzed patients

**DOI:** 10.14814/phy2.15082

**Published:** 2021-11-09

**Authors:** Vedran Premužić, Bojan Jelaković

**Affiliations:** ^1^ Department of Nephrology, Hypertension, Dialysis and Transplantation University Hospital Center Zagreb Zagreb Croatia; ^2^ School of Medicine Zagreb Croatia

**Keywords:** ambulatory, arterial stiffness, cardiovascular, hemodialysis, survival

## Abstract

We hypothesized that volume changes during 48‐h are associated with different circadian patterns of arterial stiffness on non‐dialysis day compared to dialysis day and that the night‐time increase of arterial stiffness is associated with higher mortality. The patients whose night‐time mean pulse wave velocity levels drop or rise more than 0.5 m/s compared with the day‐time period were defined as pulse wave velocity dippers and risers. The patients whose night‐time mean augmentation index drop or rise more than 10% compared with the day‐time period were defined as augmentation index dippers and risers. There was significantly higher number of patients with dipping and rising pattern of augmentation index and pulse wave velocity on non‐dialysis day when compared to dialysis day. On the non‐dialysis day both nocturnal augmentation index and pulse wave velocity levels were higher in deceased group with significantly higher number of augmentation index and pulse wave velocity dippers when compared to survived patients. In the linear regression model, the strongest association of survival was with augmentation index and pulse wave velocity risers on the non‐dialysis day. On logistic regression only pulse wave velocity rising pattern on non‐dialysis day had increased HR of 1.78 for cardiovascular mortality. The present study is the first which analyzed circadian patterns of arterial stiffness in patients on hemodialysis and compared their impact on cardiovascular mortality. A significantly greater number of patients had an augmentation index and pulse wave velocity rising pattern in the deceased group compared to the survived group of patients. Survival had the strongest association with augmentation index and pulse wave velocity risers on the non‐dialysis day.

## Graphical abstract

The present study is the first which analyzed circadian patterns of arterial stiffness in patients on hemodialysis and compared their impact on cardiovascular mortality. Significant number of patients had an augmentation index and pulse wave velocity rising pattern in the deceased group of patients. Survival had the strongest association with augmentation index and pulse wave velocity risers on the non‐dialysis day.

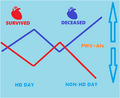

## 1 INTRODUCTION

End‐stage renal disease (ESRD) patients have highly elevated risk of cardiovascular (CV) disease at it is mostly associated with accelerated atherosclerosis, arteriosclerosis, and vascular remodeling (Ben‐Shlomo et al., 2014; Briet et al., 2012; Go et al., 2004; Vlachopoulos, Aznaouridis, O’Rourke, et al., 2010; Vlachopoulos, Aznaouridis, & Stefanadis, 2010). Decrease in arterial stiffness is connected with reduction of CV mortality not only in ESRD patients, but also in patients with different chronic kidney disease (CKD) stages (Ben‐Shlomo et al., 2014; Briet et al., 2012; Go et al., 2004; Vlachopoulos, Aznaouridis, O’Rourke, et al., 2010; Vlachopoulos, Aznaouridis, & Stefanadis, 2010). Furthermore, the nature of hemodialysis (HD) and interdialysis intervals produce shifts in volume and metabolic parameters which could additionally increase risk for future CV events especially at the first dialysis day after a long interdialytic period (Foley et al., 2011; Karpetas et al., 2015; Krishnasamy et al., 2013). The association of J‐shaped curve of blood pressure levels before and after dialysis with CV mortality is reported in some studies (Parati et al., 2016; Sarafidis, Persu, et al., 2017) but there are no present data on this pattern in arterial stiffness. Monitoring of arterial stiffness during 24‐h has become available and popular in recent years. Still, the predictive value of 24‐h monitoring of augmentation index (AIx) and pulse wave velocity (PWV) is not yet confirmed because of many limitations like lack of noninvasive reference “gold” standard, lack of reference values in ambulatory conditions, limited clinical evidence, and missing long‐term outcome studies (Omboni et al., 2016). In hemodialyzed patients ambulatory PWV and AIx are independently associated with CV events and all‐cause mortality in contrast to office blood pressure (BP) and ambulatory blood pressure monitoring (ABPM) which does not have the same prognostic use by some authors (Karpetas et al., 2015; Matschkal et al., 2019). The long interdialytic period, especially the third day has been associated with day‐ and night‐time increase in AIx and PWV as a consequence of excess fluid accumulation over the 3‐day interval which changes the function and structure of large conduit arteries independently from blood pressure and atherosclerosis. This increase of arterial stiffness markers to a volume change are a result of stiffened or noncompliant arteries (Karpetas et al., 2015). We hypothesized that volume changes during 48‐h are associated with different circadian patterns of arterial stiffness on non‐dialysis day compared to dialysis day and that the night‐time increase of arterial stiffness is associated with higher mortality. To test this hypothesis we analyzed AIx and PWV during 48‐h, compared differences between dialysis and non‐dialysis day, analyzed clinical course of HD patients, and compared circadian patterns of arterial stiffness markers between survived and deceased patients.

## 2 MATERIALS AND METHODS

In this retrospective, observational, longitudinal follow‐up study 85 hemodialyzed patients (48 men and 37 women) with a median age of 58 (21–86) years were enrolled. To minimize subjective bias in the design patients were randomly included in the study. Data on medical history and medication were collected from hospital documentation. Patients were selected if they have been on chronic hemodialysis for at least 3 months and signed informed consent. The protocols were submitted to, and approved by a local institutional review board (UHC Zagreb) in accordance with the Helsinki Declaration. When required by the IRB, written informed consent was received from every participant or legal guardian. Exclusion criteria were: atrial fibrillation or other chronic arrhythmias, stroke, transient ischemic attack (TIA) or myocardial infarction (in the three previous months), stage III‐IV of congestive heart failure, and significant hemodynamic instability during the dialysis (intradialytic hypotension: a ≥ 20 mmHg drop in systolic blood pressure or >10 mmHg drop in mean arterial pressure and the presence of symptoms related to intradialytic hypotension). Patients were on hemodialysis three times a week. All hemodialyzed patients were studied on mid‐week dialysis day after a 2‐day interdialytic interval. Ultrafiltration was calculated as a mean value of volume withdrawal based on the patients’ prespecified dry weight in last 2 weeks. Ambulatory blood pressure; systolic and diastolic blood pressure, heart rate, mean arterial pressure; hemodynamic monitoring; central systolic blood pressure and pulse pressure; and arterial stiffness; heart rate corrected AIx, and PWV, monitoring was performed using 24h Tensiomed Arteriograph (Medexpert Ltd.) on the non‐fistula arm. It was started before the mid‐week dialysis session and continued for 48 h. The device was programmed to perform readings at 15‐min intervals between 6 am and 10 pm (daytime), and 30‐min intervals between 10 pm and 6 am (nighttime). The device was also programmed to perform readings during the dialysis session at 15‐min intervals. The patients whose nighttime mean blood pressure levels drop less than 10% compared with the daytime period were defined as non‐dippers. PWV value ≥10 m/s was used as a cut‐off value according to the European Society of Hypertension/European Society of Cardiology guidelines (ESH/ESC Task Force for the Management of Arterial Hypertension, 2013). A difference of PWV of 0.5 m/s was considered as clinically meaningful (Papaioannou et al., 2012). The patients whose nighttime mean PWV levels drop or rise more than 0.5 m/s compared with the daytime period were defined as PWV dippers and risers. A difference of AIx of 10% was considered as clinically meaningful (London et al., 2001). The patients whose nighttime mean AIx drop or rise more than 10% compared with the daytime period were defined as AIx dippers and risers. The mean values of day and nighttime PWV and AIx for dialysis, and non‐dialysis days were averaged to reflect 48 h day and nighttime PWV and AIx. The 48 h PWV and AIx dippers and risers were determined the same. Follow‐up period lasted until the last enrolled patient reached the 36‐months’ time point or till the time of death.

### 2.1 Statistical methods

Statistical analysis was performed using SPSS version 23.0 (IBM Corp.). Normality of data distribution was tested using Kolmogorov–Smirnov test. Preliminary analyses were performed to ensure no violation of the assumptions of normality, linearity, and homoscedasticity. Categorical data were expressed as numbers and frequencies. Correlations were obtained using Pearson's test for normally distributed variables and Spearman rank correlation for non‐normally distributed variables. Normally distributed variables were presented as means + standard deviations and Student's *t* test for independent samples was used for comparisons between two groups. Non‐normally distributed data were presented as median and interquartile range and Mann–Whitney *U*‐test was used in comparison between two groups. Analysis of variance (ANOVA) was used to detect significant differences among ≥2 groups. Categorical variables were compared using *χ* ^2^‐test. Survival analysis was done with Kaplan–Meier curves which were tested with log‐rank test while hazard ratios were estimated with Cox proportional hazards regression. Multiple linear regression was used to explore the influence of different variables on AIx and PWV risers on non‐dialysis day, while logistic regression was used for categorical dependent variables which were showed by Forest plot. We constructed three linear regression models to assess independent associations of multiple independent variables with AIx and PWV rising pattern. A *p* value <0.05 (two‐sided tests) was considered significant.

## 3 RESULTS

### 3.1 Demographic and hemodynamic characteristics of hemodialyzed patients

A total of 85 patients on chronic hemodialysis with a median dialysis vintage of 59 (20–107) months were enrolled. Approximately one fifth of study participants had diabetes while 97.6% had hypertension. Calcium channel antagonists and β‐blockers were the most commonly prescribed antihypertensive drugs with mean of 3.37 ± 0.1 drug per patient. There were no significant differences in brachial BP, pulse pressure, heart rate, and central systolic BP between hemodialyzed patients on dialysis and non‐dialysis day while mean arterial pressure was significantly lower at night on non‐dialysis day than on dialysis day. We have not found differences in AIx and PWV values on dialysis and non‐dialysis day.

### 3.2 Circadian characteristics of blood pressure and arterial stiffness on dialysis and non‐dialysis day

Circadian characteristics of BP, AIx, and PWV values during 48 h including dialysis and non‐dialysis‐day are showed in Table 1. Significantly lower percentage of BP non‐dippers was found on non‐dialysis day when compared to dialysis day. There was significantly higher number of patients with rising pattern of AIx on non‐dialysis day when compared to dialysis day. We have found significantly higher number of patients with dipping pattern of PWV on non‐dialysis day. When we have divided hemodialyzed patients in three subgroups regarding PWV circadian pattern on the non‐dialysis day, not only PWV risers were significantly older when enrolled than PWV dippers and patients with same PWV values (*p* < 0.001), but also they were older when started with dialysis (*p* < 0.01). However, no significant differences were observed in dialysis vintage and dialytic parameters. On the non‐dialysis day patients with same PWV values and PWV risers had significantly higher ultrafiltration than PWV dippers. We have not found any significant differences regarding AIx circadian pattern. When patients were divided by sex, we have not found significant differences (all *p* < 0.05).

**TABLE 1:** Blood pressure and arterial stiffness values, and characteristics of hemodialyzed patients

	Day 48 h	Night 48 h	During dialysis	Day of dialysis day	Night of dialysis day	Day of non‐dialysis day	Night of non‐dialysis day
SBP (mmHg)	149.4 ± 3.5	148.7 ± 3.5	142.3 ± 3.4	149.7 ± 3.4	149.3 ± 3.5	150.4 ± 3.2	147.1 ± 3.6
DBP (mmHg)	84.1 ± 2.2	82.9 ± 2.2	79.2 ± 1.8^a^	83.9 ± 2.0	82.3 ± 2.0	84.5 ± 2.0	81.2 ± 2.3
MAP (mmHg)	91.0 ± 2.9	89.7 ± 2.9	90.5 ± 2.8	91.5 ± 3.0	90.6 ± 13.6	89.6 ± 2.9	88.4 ± 2.6
PP (mmHg)	83 (71–93)	82 (70–91)	80 (67–90)	83 (72–93)	84 (71–93)	82 (70–95)	81 (68–91)
HR (mmHg)	69 (57–84)	69 (58–86)	69 (59–87)	68 (54–82)	69 (59–87)	69 (55–81)	69 (56–84)
SBPL (mmHg)	155.3 ± 3.9	155.1 ± 3.9	150.2 ± 3.2	156.4 ± 3.8	154.7 ± 3.8	154.9 ± 3.8	152.4 ± 3.7
AIx (%)	37.4 ± 1.8	40.7 ± 1.8	32.5 ± 1.7^b^	37.5 ± 1.9	40.6 ± 1.8	36.7 ± 1.9	41.1 ± 1.9
PWV (m/s)	9.8 ± 0.2	9.9 ± 0.2	9.5 ± 0.2^b^	9.9 ± 0.2	9.9 ± 0.2	9.9 ± 0.2	10.0 ± 0.3

	48 h	During dialysis	Day of dialysis	Non‐dialysis day
BP Non‐dipper (*N*/%)	79 (92.9)		77 (90.6)	68 (80.0)^e^
AIx (%) (*N*)				
Dippers	6	40^c^, ^d^	3	12^e^
Same	64	37	65^c^, ^e^	38
Risers	15	8	17	35^d^, ^e^
PWV (m/s) (*N*)				
Dippers	10	59^c^	17	38^e^
Same	52	17	50^c^, ^e^	23
Risers	23	9	18	24^d^

Note

Results are shown as mean ± SD or median (interquartile range).

Abbreviations: AIx, augmentation index; DBP, diastolic blood pressure; HR, heart rate; MAP, mean arterial pressure; PP, pulse pressure; PWV, pulse wave velocity; SBP, systolic blood pressure; SBPL, systolic blood pressure load.

^a^ Statistical significance between during dialysis and day of non‐dialysis day.

^b^ Statistical significance between during dialysis and night of non‐dialysis day.

^c^ Statistical significance between day of dialysis and during dialysis.

^d^ Statistical significance between non‐dialysis day and during dialysis.

^e^ Statistical significance between dialysis and non‐dialysis day.

### 3.3 Determinants of arterial stiffness markers rising pattern on the non‐dialysis day

Regarding the obtained results on AIx and PWV circadian patterns, we have furthermore analyzed the impact of different variables on AIx and PWV rising pattern on the non‐dialysis day. In univariate analysis older age at enrollment, duration of hypertension, and intact parathyroid hormone (iPTH) were associated with AIx rising pattern while older age at enrollment, duration of hypertension, ultrafiltration, and iPTH were associated with PWV rising pattern on non‐dialysis day. In the linear regression model, except age, we have not found significant association of different variables with AIx on combined dialysis and non‐dialysis day, during dialysis, and on dialysis and non‐dialysis day. Higher PWV levels were associated with age, duration of hypertension, ultrafiltration, and iPTH on non‐dialysis day while we have not found any significant associations with different variables on combined dialysis and non‐dialysis day, during dialysis and on dialysis day. In the linear regression analysis all the results on AIx and PWV patterns are shown independent of BP levels. On logistic regression, age, and duration of hypertension had increased OR of 1.03 [CI 0.99, 1.07] and 1.01 [CI 1.004, 1.014] for AIx rising pattern on non‐dialysis day while we have not found any associations of ultrafiltration with AIx rising pattern on combined dialysis and non‐dialysis day, during dialysis and on dialysis and non‐dialysis day. Ultrafiltration, age and iPTH had increased OR of 1.01 [CI 0.96, 1.06], 1.06 [CI 1.00, 1.13], and 1.03 [CI 1.03, 1.04] for PWV rising pattern on non‐dialysis day while ultrafiltration had increased OR of 1.01 [CI 0.94, 1.08] for PWV rising pattern on combined dialysis and non‐dialysis days. We have not found associations of ultrafiltration with PWV rising pattern during dialysis or on dialysis day.

### 3.4 Differences in demographic and hemodynamic characteristics of survived and deceased hemodialyzed patients

**FIGURE 1:**
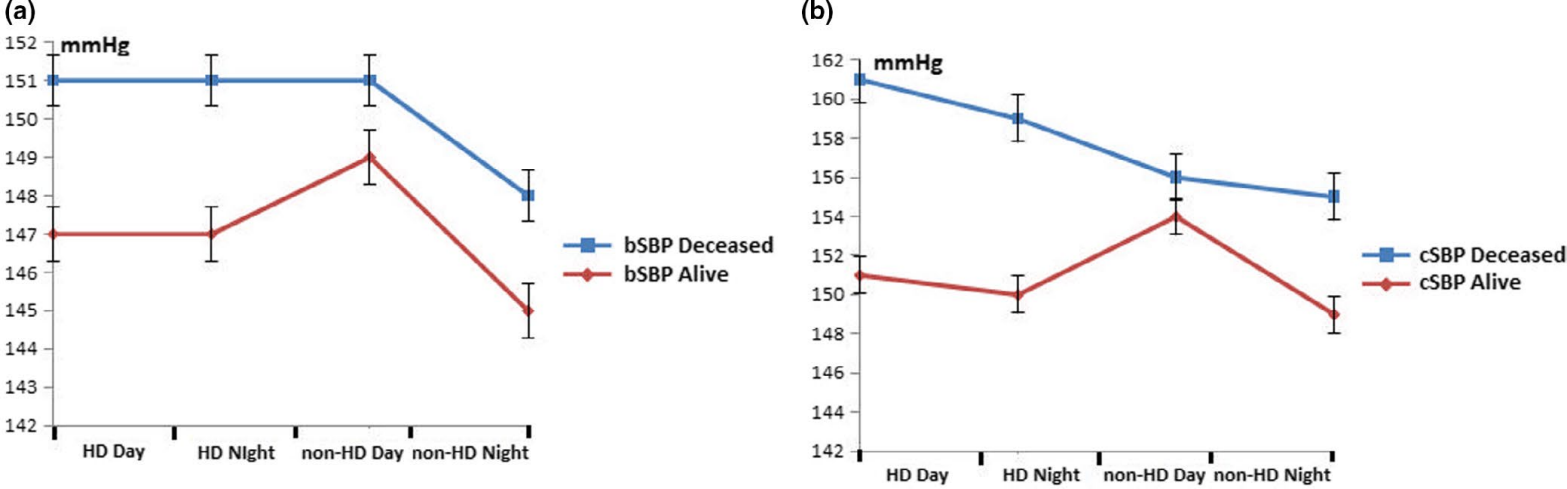
The variation of brachial systolic (a) and central systolic blood pressure (b) between deceased and survived patients during the 48 h recording. bSBP, brachial systolic blood pressure; cSBP, central systolic blood pressure; HD, hemodialysis; standard error

Patients were followed up for a total of 36 months. The incidence of CV mortality in our group of HD patients was 49.4% (men 57.1%, women 42.9%). Twelve patients have died from heart failure, 14 from stroke, 13 from myocardial infarction, and three from severe valvular disease. Deceased patients not only were older when enrolled (*p* < 0.001), but also were older when started with dialysis (*p* < 0.001) (Table 2). However, no significant differences were observed in dialysis vintage and dialytic parameters except higher ultrafiltration rates in deceased group of patients. We have not found any significant differences between deceased and survived patients in hemodynamic parameters. Figure 1 presents the variation of brachial systolic and central systolic BP between deceased and survived patients during the 48 h recording. Deceased patients had significantly higher nocturnal AIx levels and higher number of AIx and PWV risers than survived patients on combined dialysis and non‐dialysis day while we have not found differences in PWV values between these two groups of patients (Table 3).

**TABLE 2:** Demographic, clinical, and laboratory data of survived and deceased patients

	Survived (*N* = 43)	Deceased (*N* = 42)	*p*
Age (years)	51 (34–73)	68 (52–80)	**<0.001**
Males (*N*/%)	24 (55.8)	24 (57.1)	0.90*
BMI (kg/m^2^)	25.2 ± 0.7	24.9 ± 0.7	0.85
Smoker (*N*/%)	11 (25.5)	7 (16.6)	0.31*
Age on start of dialysis (years)	45 (35–56)	61 (52–71)	**<0.001**
Dialysis vintage (months)	65 (23–98)	54 (12–87)	0.44
Hypertension (Yes) (*N*/%)	41 (95.3)	42 (100.0)	0.16*
Number of antihypertensives/person	3.23 ± 0.1	3.51 ± 0.1	0.69
Duration of hypertension (months)	79.1 ± 11.4	123.3 ± 14.6	**0.02**
Diabetes (Yes) (*N*/%)	5 (11.6)	10 (23.8)	0.14*
Duration of dialysis (h)	3.8 (3.5–4.0)	3.8 (3.5–4.1)	0.63
Ultrafiltration (ml)	3034 (2600–3500)	3476 (2900–3900)	**0.01**
Kt/V	1.29 ± 0.1	1.28 ± 0.1	0.91
Residual diuresis (ml)	613 (50–1000)	413 (0–900)	0.17
Weekly vitamin D load µg/week	0.83 (0.35–0.95)	0.85 (0.36–0.98)	0.87
Daily phosphate binder calcium load (g/day)	1.6 (0.3–2.7)	1.2 (0.1–1.3)	0.28
Weekly/weight erythropoietin load (IU/kg)	113 (83–132)	117 (85–138)	0.14
Hemoglobin (g/L)	108.6 ± 1.7	103.6 ± 2.2	0.08
Serum calcium (mmol/L)	2.3 ± 0.1	2.2 ± 0.1	0.02
Serum phosphate (mmol/L)	1.5 ± 0.1	1.4 ± 0.1	0.26
iPTH (pmol/L)	26.3 (4.5–48.4)	42.7 (9.5–67.6)	0.07
Serum glucose (mmol/L)	6.2 (4.5–7.7)	7.3 (5.2–8.8)	0.17
Serum cholesterol (mmol/L)	4.1 (3.3–4.9)	4.2 (3.4–4.9)	0.69
Serum uric acid (μmol/L)	357 ± 9.5	325 ± 8.3	**0.01**
Combined dialysis and non‐dialysis day Non‐dipper (*N*/%)	40 (93.0)	39 (92.9)	0.97*
Dialysis day Non‐dipper (*N*/%)	39 (90.7)	38 (90.5)	0.97*
Non‐dialysis day Non‐dipper (*N*/%)	36 (83.7)	32 (76.2)	0.39*

Note

Results are shown as mean ± SD or median (interquartile range), categorical variables were compared using *χ* ^2^‐test*. *P* values indicate bold significant.

Abbreviations: BMI, body mass index; CV, cardiovascular; iPTH, intact parathyroid hormone.

**TABLE 3:** Arterial stiffness characteristics of survived and deceased patients

	Survived (*N* = 43)	Deceased (*N* = 42)	*p*
Day 48 h AIx (%)	37.0 ± 2.7	37.2 ± 2.4	0.85
Night 48 h AIx (%)	36.4 ± 2.5	45.2 ± 2.5	**0.02**
48 h AIx (%) Risers (*N*/%)	7 (16.3)	16 (38.1)	0.02*
Day 48 h PWV (m/s)	9.7 ± 0.3	9.8 ± 0.2	0.87
Night 48 h PWV (m/s)	9.6 ± 0.3	10.3 ± 0.3	0.13
48 h PWV (m/s) Risers (*N*/%)	4 (9.3)	18 (42.8)	<0.001*
Day of dialysis Day AIx (%)	36.8 ± 2.7	38.2 ± 2.6	0.72
Night of dialysis Night AIx (%)	36.6 ± 2.7	44.6 ± 2.3	**0.02**
Day of dialysis AIx (%) Risers (*N*/%)	5 (11.6)	14 (33.3)	0.01*
Day of dialysis Day PWV (m/s)	9.9 ± 0.3	9.8 ± 0.2	0.79
Day of dialysis Night PWV (m/s)	9.6 ± 0.3	10.3 ± 0.3	0.12
Day of dialysis PWV (m/s) Risers (*N*/%)	2 (4.6)	16 (38.1)	<0.001*
During dialysis AIx (%)	32.0 ± 2.4	33.5 ± 2.7	0.69
During dialysis AIx (%) Same/Risers (*N*/%)	2 (4.6)	2 (4.7)	0.97*
During dialysis PWV (m/s)	9.3 ± 0.3	9.6 ± 0.2	0.39
During dialysis PWV (m/s) Same/Risers (*N*/%)	18 (41.9)	28 (66.6)	0.02*
Day of non‐dialysis Day AIx (%)	38.4 ± 2.8	35.0 ± 2.6	0.39
Day of non‐dialysis Night 48 h AIx (%)	33.8 ± 2.6	48.6 ± 2.4	<0.001*
Day of non‐dialysis AIx (%) Risers (N/%)	7 (16.3)	34 (80.9)	<0.001*
Day of non‐dialysis Day PWV (m/s)	9.9 ± 0.3	9.8 ± 0.3	0.73
Day of non‐dialysis Night PWV (m/s)	9.4 ± 0.3	10.6 ± 0.3	**<0.01**
Day of non‐dialysis PWV (m/s) Same/Risers (N/%)	1 (2.3)	36 (85.7)	<0.001*
PWV≥10 m/s (N/%)	14 (32.5)	21 (50.0)	0.10

Note

Results are shown as mean ± SD or median (interquartile range), categorical variables were compared using *χ* ^2^‐test*. *P* values indicate bold significant.

Abbreviations: AIx, augmentation index; PWV, pulse wave velocity.

### 3.5 Association of cardiovascular mortality with circadian characteristics of arterial stiffness

**FIGURE 2:**
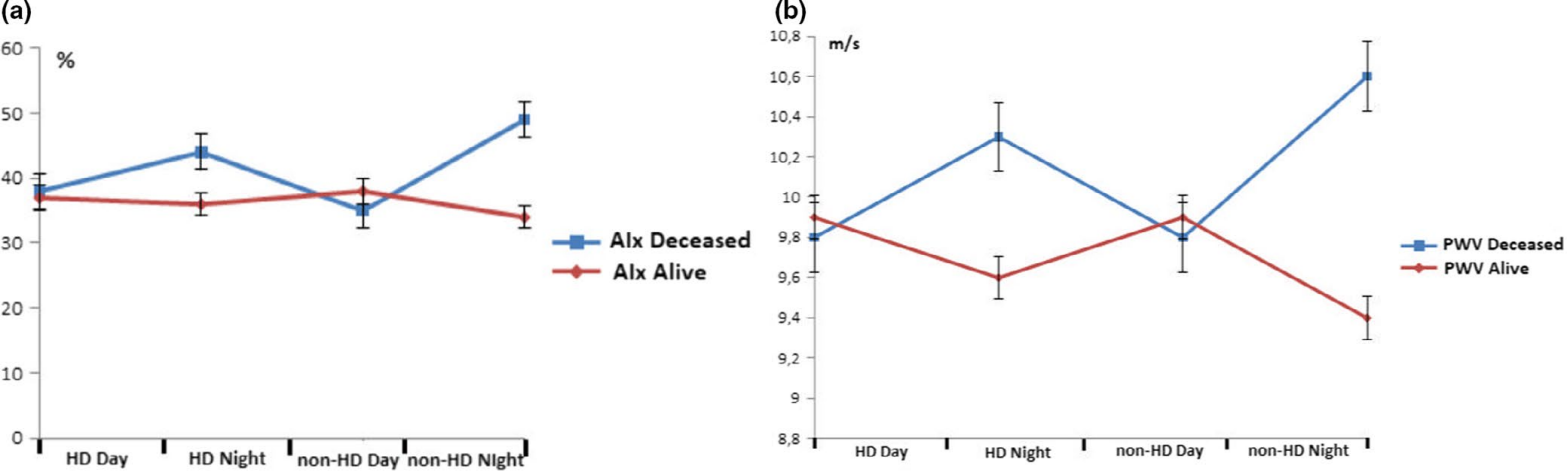
The variation of augmentation index (a) and pulse wave velocity (b) between deceased and survived patients during the 48 h recording. AIx, augmentation index; HD, hemodialysis; PWV, pulse wave velocity; standard error

**FIGURE 3:**
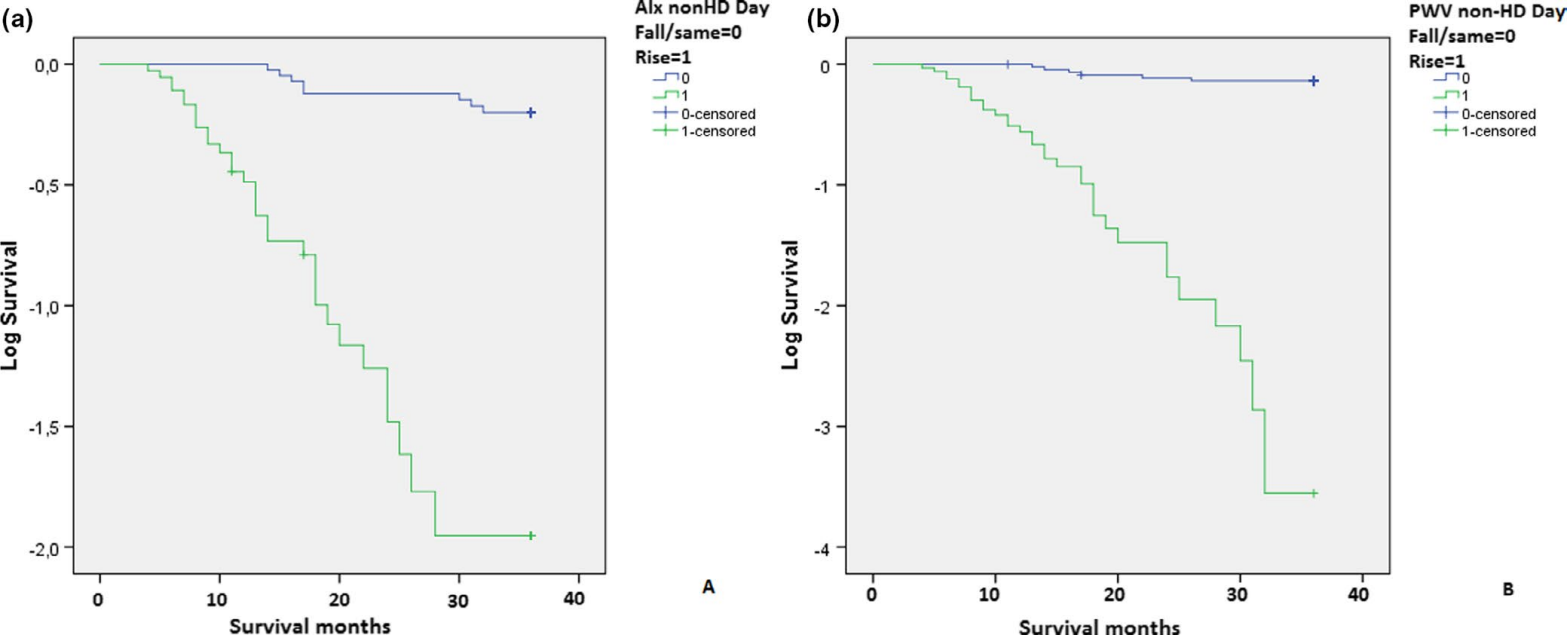
Cardiovascular mortality in AIx non‐risers and risers (a) and PWV non‐risers and risers on non‐HD day at the end of follow‐up. AIx, augmentation index; HD, hemodialysis; PWV, pulse wave velocity

**FIGURE 4:**
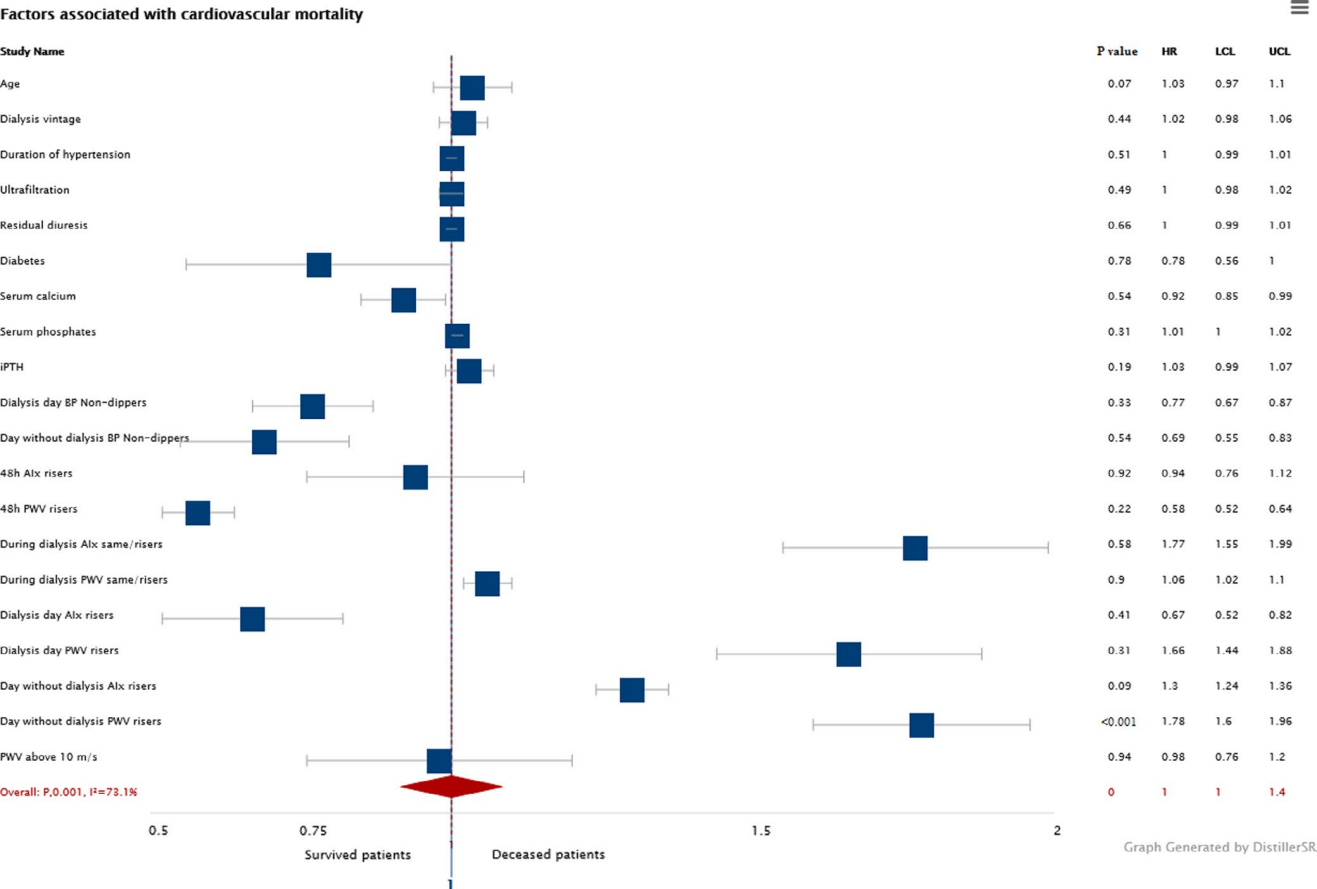
Forrest plot for cardiovascular mortality in HD patients. AIx, augmentation index; BP, blood pressure; HD, hemodialysis; iPTH, intact parathyroid hormone; PWV, pulse wave velocity

We have found the same AIx and PWV nocturnal pattern in the dialysis day between deceased and survived patients with significantly higher number of AIx and PWV risers in the deceased group of patients. During the dialysis, deceased patients had significantly higher number of PWV risers and same levels than survived patients. On the non‐dialysis day, both nocturnal AIx and PWV levels were higher in deceased group with significantly higher number of AIx and PWV dippers when compared to survived patients. Figure 2 presents the variation of AIx and PWV during the 48 h recording between deceased and survived patients on hemodialysis. When we have divided hemodialyzed patients in two subgroups regarding AIx circadian pattern in non‐dialysis day, we have not found any significant differences except AIx risers were older when enrolled and older when started with dialysis and survived shorter than AIx non‐risers. When we have divided them in two subgroups regarding PWV circadian pattern on the non‐dialysis day, PWV risers were significantly older when enrolled and older when started with dialysis and had significantly higher ultrafiltration rates than PWV non‐risers and survived significantly shorter. Mean survival time was longer in combined dialysis and non‐dialysis day AIx and PWV non‐risers (*p* < 0.01; *p* < 0.001); day of dialysis AIx and PWV non‐risers (*p* < 0.01; *p* < 0.001); and non‐dialysis day AIx and PWV non‐risers (*p* < 0.001; *p* < 0.001; Figure 3) than in AIx and PWV risers. In the linear regression model (Table 4), survival was negatively associated with age, duration of hypertension, and ultrafiltration while the strongest association was with AIx and PWV risers on the non‐dialysis day. Importantly, combined dialysis and non‐dialysis day and dialysis day AIx and PWV risers have not been associated with survival in our group of patients. On logistic regression, only PWV rising pattern on non‐dialysis day, while interestingly not age, duration of hypertension, or AIx rising pattern, had increased HR of 1.78 [CI 1.60, 1.96] for CV mortality (Figure 4).

**TABLE 4:** Factors associated with survival and cardiovascular mortality

	Linear regression analysis	*p*	Cox regression analysis	*p*
	Beta		Adjusted HR	
Age	−.382	**0.01**	1.03	0.07
Dialysis vintage	−.021	0.86	1.02	0.44
Duration of hypertension	−.230	0.07	1.00	0.51
Ultrafiltration	−.313	**0.02**	1.00	0.49
Residual Diuresis	−.130	0.33	1.00	0.66
Diabetes	−.002	0.98	0.78	0.78
Serum calcium	.090	0.45	0.92	0.54
Serum phosphate	.027	0.83	1.01	0.31
iPTH	−.136	0.27	1.03	0.19
Dialysis Day BP Non‐Dippers	.064	0.53	0.77	0.33
Non‐dialysis Day BP Non‐Dippers	.012	0.89	0.69	0.54
48 h AIx Risers	−.111	0.26	0.94	0.92
48 h PWV Risers	−.048	0.60	0.58	0.22
During dialysis AIx Same/Risers	.113	0.19	1.77	0.58
During dialysis PWV Same/Risers	.021	0.79	1.06	0.90
Dialysis Day AIx Risers	.173	0.11	0.67	0.41
Dialysis Day PWV Risers	.021	0.83	1.66	0.31
Non‐dialysis Day AIx Risers	−.569	**<0.001**	1.30	0.09
Non‐dialysis Day PWV Risers	−.365	**<0.01**	1.78	**<0.001**
PWV≥10 m/s	−.055	0.45	0.98	0.94

*P* values indicate bold significant.

Abbreviations: AIx, augmentation index; BP, blood pressure; iPTH, intact parathyroid hormone; PWV, pulse wave velocity.

## 4 DISCUSSION

This is the first study to perform 48‐h ambulatory monitoring of arterial stiffness parameters, AIx and PWV, in hemodialyzed group of patients with the 24‐h Arteriograph device. The main finding of this study was reported different circadian patterns of ambulatory monitored AIx and PWV in hemodialyzed patients and showed high percentage of patients with rising pattern. The differences between different PWV circadian patterns were mostly seen on the non‐dialysis day. Furthermore, we have found significantly higher number of AIx and PWV risers in the deceased group of patients in combined dialysis and non‐dialysis day and both dialysis and non‐dialysis day. Survival had the strongest association with AIx and PWV risers on the non‐dialysis day. HR for CV mortality was solely significantly increased for patients with circadian PWV rising pattern on the non‐dialysis day while interestingly age was not associated with CV mortality.

Our data on AIx and PWV reduction during dialysis are in accordance with previous results (Di Iorio et al., 2010; Karpetas et al., 2015), and shows the association with changes and reduction of hydration status. Although some reports suggested that AIx reduction is more pronounced in acute changes following hydration status during dialysis (Laurent et al., 2006) and PWV response differently without significant changes due to long‐term structural changes in large artery walls (Lin et al., 2005), our study showed that both smaller arteries and large arteries, which are more sustainable to rapid volume changes, responds with elevation of pressure waveforms. This finding could easily be confirmed with our results on higher percentage of patients with rising AIx and PWV pattern on the non‐dialysis day where the fluid accumulation is the highest at the end of interdialytic period just before another hemodialysis session. Strict volume control and individualized prescription of ultrafiltration is associated with reduction of both AIx and PWV (Georgianos et al., 2013; Hur et al., 2013; Lin et al., 2003). This fluid accumulation is mostly responsible for elevated risk of fatal CV events in the last 12‐h period of the interdialytic interval (Bleyer et al., 1999; Karnik et al., 2001). Nevertheless, we have not found significant differences in AIx and PWV between dialysis and non‐dialysis day. Although several studies reported the impact of weight gain on circadian ambulatory blood pressure pattern during the interdialytic period we have not found this association (Agarwal, 2009; Agarwal & Light, 2008). Non‐dialysis day even showed a decrease in number of BP non‐dippers when compared to dialysis day while arterial stiffness raised inversely. Interestingly, BP non‐dipping pattern did not mimic arterial stiffness patterns but on the contrary was the opposite. These changes are not only consistent with accelerated atherosclerosis and earlier vascular aging which could be seen through association of AIx and PWV risers with the duration of hypertension, but also with the higher ultrafiltration rates during dialysis as a direct result of interdialytic weight gain. Therefore, the obtained results which showed that rising AIx and PWV pattern is mostly pronounced on the non‐dialysis day is an indirect proof that more frequent dialysis sessions with smaller ultrafiltration rates could play a beneficial role in reducing CV risk in hemodialyzed patients.

Prognostic value of increased PWV in ESRD patients is confirmed in different studies (Guerin et al., 2001; Verbeke et al., 2011) while some tried to introduce a pathological PWV cut‐off value in HD patients associated with increased mortality (Blacher et al., 2003; Marshall et al., 2015; Mimura et al., 2005). Our results did not show this association. We have not found difference in survival between patients with pathological PWV ≥10 m/s as well as we have not found differences between different PWV quartiles in mean survival. These results could be observed due to the fact that different studies have measured office PWV on various time related to dialysis, before, during, or after dialysis session or on the non‐dialysis day. Furthermore, our results confirmed recent data on superiority of ambulatory 24‐h monitoring over office measurement of AIx and PWV for CV mortality predictivity in HD patients (Karpetas et al., 2015; Matschkal et al., 2019; Sarafidis, Loutradis, et al., 2017). Although we have not found significant differences in AIx and PWV between dialysis and non‐dialysis day, our results on higher percentage of patients with rising AIx and PWV pattern on the non‐dialysis day where the fluid accumulation is the highest at the end of interdialytic period just before another hemodialysis session, strong association in linear regression analysis of both AIx and PWV risers on the non‐dialysis day and increased HR only for PWV risers on the non‐dialysis day is in accordance with these studies. CV mortality in our group of HD patients was, as expected, associated with age and with PWV risers on non‐dialysis day but interestingly there was no difference in blood pressure dipping pattern between deceased and survived patients. This is in according with studies which reported association of ambulatory PWV and AIx with CV events and all‐cause mortality in HD patients in contrast to office BP and ABPM which does not have the same prognostic use (Karpetas et al., 2015; Matschkal et al., 2019).

In contrast with previous studies, we have not found differences in ambulatory combined dialysis and non‐dialysis day or dialysis and non‐dialysis day values of AIx and PWV between deceased and survived HD patients but, at least to our knowledge, this is the first study which reported different circadian patterns of ambulatory monitored AIx and PWV in HD patients and its impact on CV mortality. Our results showed that rising AIx and PWV pattern is significantly more pronounced in deceased patients when analyzing both dialysis and non‐dialysis day. The reason why only PWV risers and not AIx risers were associated with increased CV mortality could be explained through changes in AIx which are more pronounced in rapid fluid removal during dialysis (Laurent et al., 2006) while with gradual fluid accumulation during non‐dialysis day these changes are not present. These results expands the prognostic role of ambulatory 48‐h monitoring of AIx and PWV in HD patients, by showing that increasing night‐time AIx and PWV on the non‐dialysis day was independently associated with shorter survival while only increasing nigh‐time PWV on the non‐dialysis day displayed markedly higher HR for CV death.

This work has some limitations. First, 24‐h arteriograph records ambulatory central systolic BP, AIx and PWV by an oscillometric method. There is no present validation study regarding this device in patients on hemodialysis without the presence of reference values in ambulatory conditions. All current devices for ambulatory monitoring of arterial stiffness have limited clinical evidence and results should be observed with precaution. Second, we have enrolled patients from only one dialytic unit, another possible limitation is that our sample size was probably too small and the follow‐up period was relatively short. Third, unfortunately we did not perform an echocardiogram (ECHO), measured carotid intima media thickness or determine albuminuria as markers for target organ damage.

In conclusion, this study is the first which analyzed circadian patterns of arterial stiffness in patients on hemodialysis and compared their impact on CV mortality. Significant number of patients had an AIx and PWV rising pattern in the deceased group of patients. Survival had the strongest association with AIx and PWV risers on the non‐dialysis day. Future prospective studies are needed for explaining the impact of different arterial stiffness circadian patterns in hemodialyzed patients on CV outcome, the importance of rising the number of dialysis sessions per week and probably more gentle fluid removal per session. This study showed a superiority of ambulatory arterial stiffness monitoring over office measurement in prediction of CV mortality especially on non‐dialysis day.

## CONFLICT OF INTEREST

The authors declare no conflict of interest.

## AUTHOR CONTRIBUTION

All authors made substantial contributions to the conception and design of the work, analysis and or interpretation of data, drafted the work, and revised it critically for important intellectual content, and approved the version to be published.
